# Advances in the roles and mechanisms of lignans against Alzheimer’s disease

**DOI:** 10.3389/fphar.2022.960112

**Published:** 2022-10-12

**Authors:** Na Han, Yuanyuan Wen, Zhihui Liu, Jianxiu Zhai, Sikai Li, Jun Yin

**Affiliations:** Development and Utilization Key Laboratory of Northeast Plant Materials, School of Traditional Chinese Materia, Shenyang Pharmaceutical University, Shenyang, China

**Keywords:** lignans, Alzheimer’s disease, nerve protection, mechanism, dibenzocyclooctene lignans

## Abstract

**Background:** Alzheimer’s disease (AD) is a serious neurodegenerative disease associated with the memory and cognitive impairment. The occurrence of AD is due to the accumulation of amyloid *β*-protein (Aβ) plaques and neurofibrillary tangles (NFTs) in the brain tissue as well as the hyperphosphorylation of Tau protein in neurons, doing harm to the human health and even leading people to death. The development of neuroprotective drugs with small side effects and good efficacy is focused by scientists all over the world. Natural drugs extracted from herbs or plants have become the preferred resources for new candidate drugs. Lignans were reported to effectively protect nerve cells and alleviate memory impairment, suggesting that they might be a prosperous class of compounds in treating AD.

**Objective:** To explore the roles and mechanisms of lignans in the treatment of neurological diseases, providing proofs for the development of lignans as novel anti-AD drugs.

**Methods:** Relevant literature was extracted and retrieved from the databases including China National Knowledge Infrastructure (CNKI), Elsevier, Science Direct, PubMed, SpringerLink, and Web of Science, taking lignan, anti-inflammatory, antioxidant, apoptosis, nerve regeneration, nerve protection as keywords. The functions and mechanisms of lignans against AD were summerized.

**Results:** Lignans were found to have the effects of regulating vascular disorders, anti-infection, anti-inflammation, anti-oxidation, anti-apoptosis, antagonizing NMDA receptor, suppressing AChE activity, improving gut microbiota, so as to strengthening nerve protection. Among them, dibenzocyclooctene lignans were most widely reported and might be the most prosperous category in the develpment of anti-AD drugs.

**Conclusion:** Lignans displayed versatile roles and mechanisms in preventing the progression of AD in *in vitro* and *in vivo* models, supplying potential candidates for the treatment of nerrodegenerative diseases.

## 1 Introduction

Over the past 200 years, the development in public health and medical interventions had led to longer life expectancy, and population ageing has gradually become a challenge that couldn’t be ignored in the social and economic development. The global burden of pathological diseases related to aging is steadily increasing. Alzheimer’s disease (AD) was considered to be the most common age-related neurodegenerative disease and one of the most serious problems facing the world’s aging population (Ermogenous et al., 2020). The World Health Organization estimated that more than 55 million people worldwide suffered from dementia, with nearly 10 million new cases each year. The global patients of dementia were expected to reach 82 million by 2030 and 152 million by 2050 (Organization, 2021).

At present, it is generally accepted that typical histopathological traits of AD are the aggregation of amyloid *β*-protein (Aβ) in senile plaques and neurofibrillary tangles (NFTs) of tau protein. However, the perception of AD are far from enough and the treatments are still limited. Untill now, there weren’t a drug completely curing AD or reversing the progression. Donepezil, rivastigmine, galanthamine and memantine were four medicines approved by FDA in which the first three were acetylcholinesterase inhibitors (AChEI) (Ali et al., 2015; Behl et al., 2022) and memantine was an antagonist of N-methyl-D-aspartate receptor (NMDAR) (Sonkusare et al., 2005). They were usually used in alone or combination in the clinic according to the conditions of patients, and some were reported to cause hepatotoxicity, gastrointestinal-related adverse reactions and muscle-related adverse reactions, which might lead to acute renal failure secondary to rhabdomyolysis in severe cases (Ali et al., 2015; Weller and Budson, 2018). Adduhelm (Aducanumab) is a monoclonal antibody that could selectively combine with the amyloid protein in brain and reduce the deposition of Aβ plaques in neurons (Sevigny et al., 2016). It is the first drug targeting the deposition of Aβ plaques approved by FDA in 2021 and used in treating early Alzheimer’s disease. However, clinical trials revealed that about 30%–40% of the patients taking adduhelm appeared brain microbleeds and edema (Torre and Lima., 2021). Sodium Oligomannate Capsules (GV-971) was a medicine affecting the neurological function by targeting the brain-gut axis. It was approved to treat mild and moderate Alzheimer’s disease in China in 2019. The side effects were rare due to its natural character (oligosaccharide from Alge), but more clinical observations were needed to evaluate its long-term effects (Luo, 2020). The demand of anti-AD drugs with better effects or lower side effects are still in increasing, however, the success rate of drug development against AD was very low, 99% of the candidate drugs were in failure (Tatulian, 2022).

**FIGURE 1:**
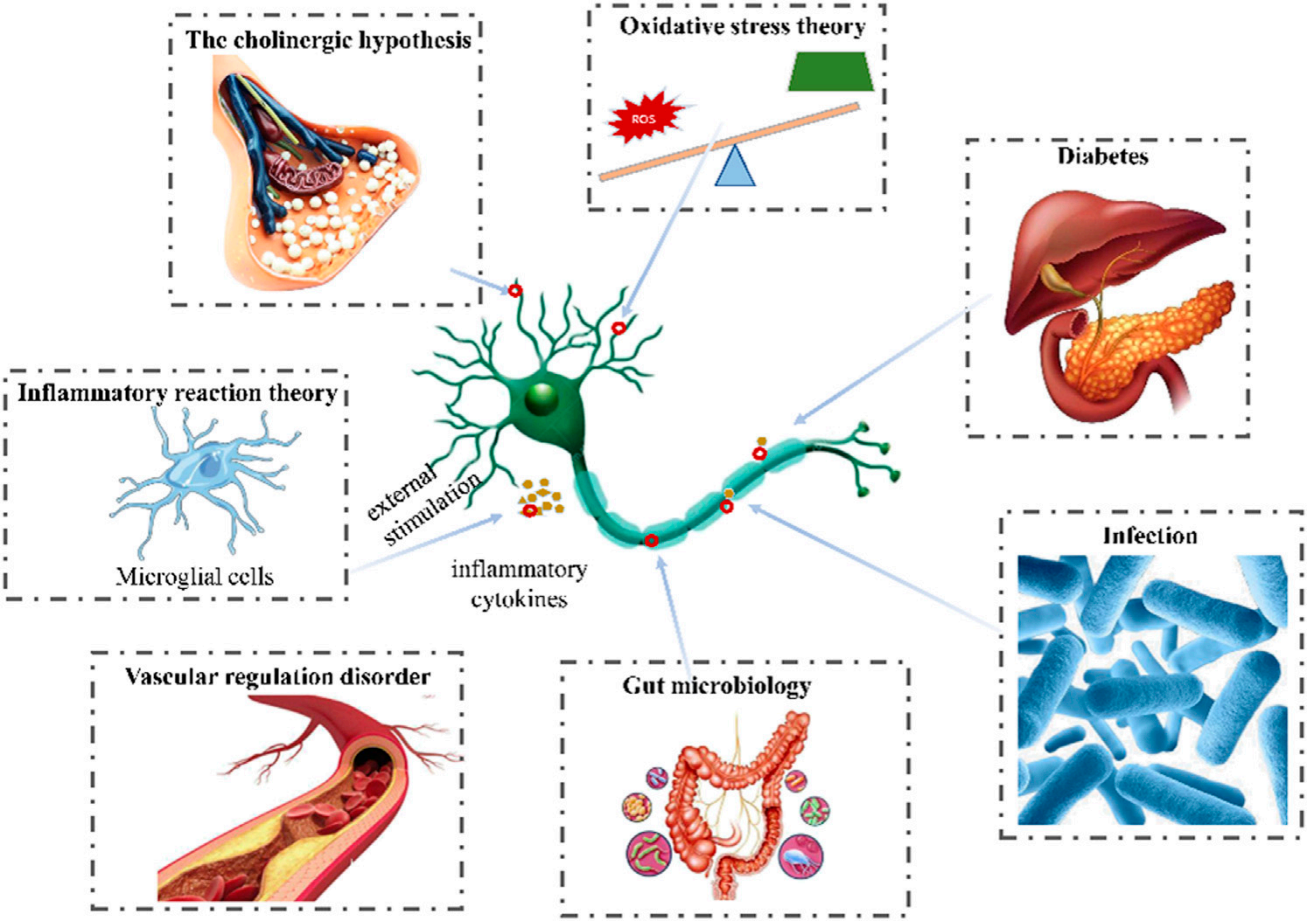
Pathogenesis of AD (The pathogenesis of AD include vascular regulation disorder, infection, diabetes, inflammatory, oxidative stress, the cholinergic hypothesis, glutamic acid pathway and gut microbiology.).

**FIGURE 2:**
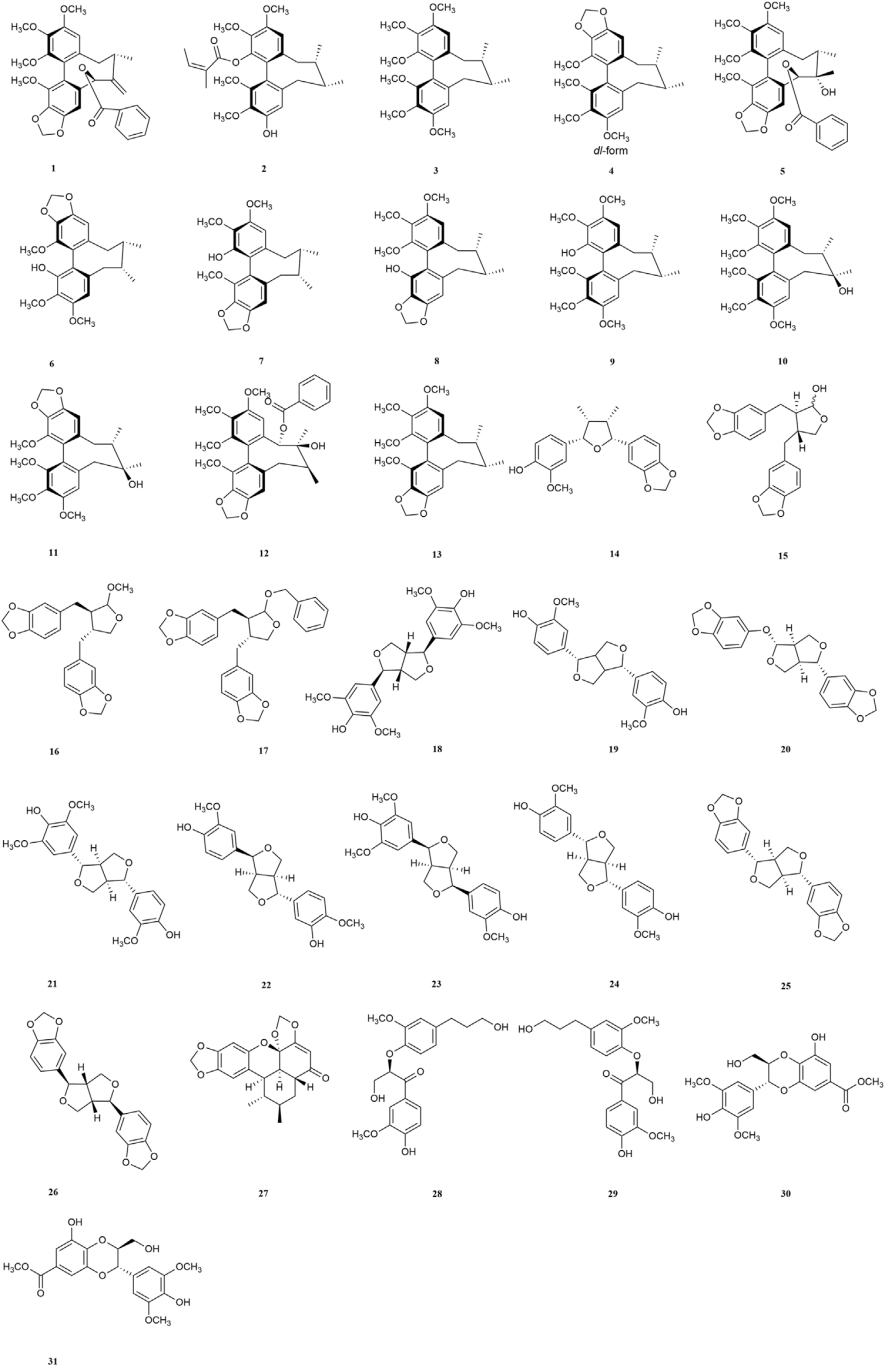
Structure of compounds **1–31**.

Plant chemicals might constitute important resources in the development of anti-AD due to their neuroprotective activities (Vaiserman et al., 2020). In the past, lignan and its extract were reported to effectively protect neuronal cell and improve cognitive ability (Zhou et al., 2021), however, their roles and mechanisms had never been summerized before. In this review, we referred the researches on the functions and mechanisms of lignans as well as the pathogenesis of AD, and analyze the potentiality of lignans in treating neurodegenerative diseases, aiming to supplying more candidates for anti-AD agents in the future. The pathogenesis of AD and structures of lignans in this paper are shown in Figure 1 and Figure 2.

## 2 Pathogenesis of Alzheimer’s disease

### 2.1 Vascular regulation disorder

Increasing evidences suggested that the vascular dysregulation made the risk of AD increased (Lee et al., 2020). Elderly patients with AD are usually accompanied by cerebrovascular diseases, such as cerebral ischemia, hypoxia and hypoperfusion (Cipollini et al., 2020). Aβ deposition was reported to be observed in cerebral parenchyma and cerebral vessels in AD (Guo et al., 2020). And *β*-secretase protein level and enzyme activity in cerebrospinal fluid were increased, which is the key rate-limiting protease to produce Aβ (Hardy and Selkoe, 2002). According to the two-hit vascular hypothesis of AD (Nelson et al., 2016), cerebrovascular damage (hit 1) is an initial insult that directly initiates neuronal injury and neuro degeneration as well as promotes accumulation of Aβ toxin in the brain (hit 2). It was reported that a large number of inflammatory mediators were released to induce neuronal apoptosis and other neurological diseases during cerebral ischemia reperfusion (Cao et al., 2015). Vascular dysfunction would also induce the dimishment of Aβ clearance and increase of its production by influencing the amyloidogenic pathway in the brain (Zlokovic, 2011; Nelson et al., 2016).

### 2.2 Infection

Microbial infection is considered as an important cause in the occurence of AD. Sureda et al. detected gingipain (an enzyme secreted by *Porphyromonas gingivalis*) in the brain of mild to late AD patients (Sureda et al., 2020). Another example is the bacterial functional amyloid known as curli secreted by *Escherichia coli* migrate in the brain and triggers AD (Zhan et al., 2016). Bacteria could infect or colonize different cells in the brain, such as microglia, inducing AD (Bu et al., 2015). Besides, the bacteria and their components (capsular proteins, flagellin, fimbrillin, peptidoglycan, proteases) were considered as the pathogen-associated molecular patterns (PAMPs) and could activate immune cells and interact with pattern recognition receptors (PRRs) such as TLR-2 and TLR-4, resulting in the pro-inflammatory cytokine secretion. This could result in a neuroinflammatory state leading to neuronal destruction and the disruption of the blood-brain barrier (BBB), promoting Aβ deposition (Zlokovic, 2011; Keaney and Campbell, 2015).

### 2.3 Type 2 diabetes

Type 2 diabetes is the most common endocrine and metabolic disease, which is caused by insulin deficiency or insulin resistance. It has been reported to induce significant cognitive decline, leading to AD, especially in elderly patients (Ding et al., 2010). Insulin could cross the blood-brain barrier, and combine with insulin receptors of many brain cells (Banks et al., 2012). However, impaired insulin signaling and insulin resistance made the expression of insulin degrading enzyme (IDE) reduced in brain (Mullins et al., 2017), which is a significant contributor to Aβ degradation (Jayaraman and Pike, 2014). The downregulation of IDE could lead to the decrease of Aβ clearance and subsequently increase of Aβ accumulation in the brain as well as tau phosphorylation (Jolivalt et al., 2010).

### 2.4 Inflammatory reaction theory

Neuroinflammation is an immune response activated by glial cells in the central nervous system, which is considered to be one of the reasons causing kinds of neurodegenerative diseases including AD. Microglia are the brain’s innate immune cells, making up about 10% of all cells in the central nervous system. In AD patients, amyloid *β*-protein could combine with the receptors of microglia cells and then induce the release of inflammatory cytokines or chemokines such as COX-2, TNF-α, ILs, iNOS and so on, further impair the cognitive function. Therefore, the inhibition against activation of microglia cells, inflammatory factors, or downregulation the inflammation signaling pathways would all excert neroprotection functions. There are many signaling pathways involving in the regulation of neuroinflammation, in which P38/MAPK pathway, JNK/MAPK pathway, PI3K/AKT/GSK-3β/NRF2 pathway, MyD88 pathway, NF-κB pathway are all widely reported (Munoz and Ammit, 2010; Cuadrado et al., 2018; Fao et al., 2019; Ju Hwang et al., 2019).

### 2.5 Oxidative stress theory

Oxidation stress is closely related to the early occurcence of AD and its progression. Overproduced reactive oxygen species (ROS) could promote the secretion of inflammatory factors, form a cascade reaction to expand inflammation, and promote the pathological process of AD (Christov et al., 2004). Besides, extra ROS would also lead to mitochondrial damage (Sorce et al., 2017), which in turn activated NADPH oxidase to generate ROS again, aggravated the occurrence of oxidative stress (Sorce et al., 2017; Zott et al., 2018), causing protein and DNA dysfunction, and finally nerve cells apoptosis (Eckert et al., 2008; Yaribeygi et al., 2018). They could also bind to lipids and proteins of nerve cells to induce lipid peroxidation reaction, destroying the stability and fluidity of the membrane, leading to cell apoptosis (Morris, 2012). Thus, antioxidants or those with abilities to stimulate the oxidation defense system including enzymatic and nonenzymatic groups such as superoxide dismutase (SOD), malondialdehyde catalase (CAT), glutathione (GSH) etc. might be useful in blocking the occurcence or progression of AD.

### 2.6 The cholinergic hypothesis

Early in the 1970s, Deutsch et al. have found that cholinergic systems were associated with memory formation and storage (Deutsch, 1971). Acetylcholine (Ding et al., 2010; Whitmer et al., 2021) is an important central excitatory neurotransmitter responsible for cognitive function and learning (Wang and Zhang, 2019). The function of cholinergic system in AD patients was defective (Lannfelt et al., 1993). Nucleus basalis of meynert (NBM) is the main distribution area of cholinergic neurons, which were reported to lost and degenerated in AD patients according to morphological studies (Briggs et al., 1997). Further study found that the concentration of Ach was significantly reduced in the brain of patients, which accelerated Aβ deposition, and induced a variety of pathological phenomena such as abnormal phosphorylation of Tau protein, neuronal inflammation, apoptosis, imbalance of neurotransmitter and neurohormone systems (Murray et al., 2013; Stepanichev et al., 2014). Acetylcholinesterase (AchE) is the decomposition enzymes of Ach, which could catabolize Ach into inactive choline and acetic acid metabolites and often used to evaluate the activity of cholinergic nervous system (Khan et al., 2018). In addition, AchE also participated in the formation of amyloid protein in brain cells (Houghton et al., 2006). Due to the role in the development of amyloid protein and the hydrolysis of Ach, the inhibition of AchE is regarded as a promising strategy for the treatment of AD.

### 2.7 Glutamatergic hypothesis

Impairment of the glutamatergic system is widely considered to be associated with pathomechanisms of AD (Danysz and Parsons, 2012). Many studies have shown that glutamate signaling pathway played an important role in synaptic plasticity and is dysregulated in AD (Taniguchi et al., 2022). Functional n-methyl-d-aspartate (NMDA) channels are heteromeric tetramers of GLUN1 and GLUN2A-D subunits. GluN2B containing NMDA receptors account for about 50% of all NMDA receptors (Chazot and Stephenson, 1997). In the different subtypes of NMDA receptors, GLUN2B types are the most prominent in the forebrain, which provided superior treatment target for AD (Yashiro and Philpot, 2008). When soluble Aβ oligomers (AβOs) binds to these receptors such as NMDA receptors on the cell membrane, it will cause neurotoxicity and AD (Cline et al., 2018).

### 2.8 Gut microbiota

Recent studies have shown that the pathogenesis of many neurodegenerative diseases may be related to intestinal flora. Symbiotic flora in the gastrointestinal tract regulate the neuroinflammation and central nervous system autoimmunity through gut-brain axis (La Rosa et al., 2018). Researchers found that the most distinctive changes of gut microflora in AD patients were the decreasing of *Bifidobacterium breve* strain A1 and the increasing of Firmicutes and Bacteroidetes, which could lead to the enhancement of the inflammation levels in the plasma and brain (Bostanciklioglu, 2019). Clinical studies have shown that *Bifidobacterium breve* strain A1 is benificial to improve cognitive and mental health in patients with mild cognitive impairment (Okubo et al., 2021). The increasing of Firmicutes and Bacteroidetes would lead to the decrease of cognitive function in AD patients (Wu et al., 2020).

## 3 Lignans as candidate phytochemicals

### 3.1 Dibenzocyclooctene lignans

#### 3.1.1 Schisandrin

Hu et al. reported that eleven dibenzocyclooctene lignans **(1–11)** from the fruits of *Schisandra chinensis* had inhibitory effects on LPS-induced NO release in mouse BV2 microglial cells. It was worth noting that schisandrin **(10)** had the best activity (Hu et al., 2014), supplying a very prosperous candidates in treating AD. Besides, *S*-biphenyl as well as methylenedioxy were found to be the active groups according to structure-activity relationship studies, however, the presence of acetyl group on cyclooctadiene or hydroxyl group on C-7 would reduce the inhibitory activity on NO release (Hu et al., 2014).

In the *in vivo* model, Hu et al. also found that schisandrin **(10)** could significantly improve the short-term and spatial reference memory impairment of mice induced by Aβ. Glutathione peroxidase (GSH-Px) activity, GSH content in the cerebral cortex and hippocampus of rats increased, while MDA and GSSG contents decreased (Hu et al., 2012), suggesting schisandrin (**10**) might improve cognitive impairment through antioxidation.

#### 3.1.2 Gomisin A

Gomisin A (**11**) in the fruits of *Schisandra chinensis* was reported not only to inhibit the production of NO, PGE2, but also suppress the expressions of iNOS and COX-2 in LPS-stimulated N9 microglia. TLR-4 is one of the main receptors in microglia and gomisin A (**11**) was reported to attenuate microglia-mediated neuroinflammation by inhibiting TLR-4-mediated NF-κB and MAPKs signaling pathways. Moreover, it also significantly inhibited LPS-induced ROS, NADPH oxidase activation, and GP91phox expression in microglia (Wang et al., 2014). At the same time, it reduced mRNA expression and pro-inflammatory cytokines TNF-α, IL-1β and IL-6 production (Dong et al., 2006).

#### 3.1.3 Schisantherin A

Schisantherin A (**12**) is a major bioactive lignan isolated from the fruits of *Schisandra chinensis,* which has potential therapeutic value for neurodegenerative diseases related to abnormal oxidative stress. In the *in vivo* studies, schisantherin A (**12**) increased the activities of SOD and GSH-Px, decreased the contents of Aβ and activities of MDA in hippocampus and cerebral cortex. It also significantly decreased the histopathological changes in the hippocampus (Li et al., 2014). Zhang et al. found that schisantherin A (**12**) could down-regulate the expression of iNOS, the accumulation of ROS, and inhibit the excessive production of NO in SH-SY5Y cells induced by 6-OHDA (Zhang et al., 2015). Results showed that schisantherin A (**12**) might exert neroprotection effects by both anti-inflamation and anti-oxidation.

#### 3.1.4 Schisandrin B

In the *in vivo* study, Schisandrin B (SchB) (**13**) could significantly suppress the AChE activity and increase the level of Ach in scopolamine-induced dementia mice model (Giridharan et al., 2011). In differentiated neuronal PC12 cells of rats exposed to 3-nitropropionic acid (3-NP), SchB showed the ability to resist apoptosis and necrosis by blocking the JNK-mediated pyruvate dehydrogenase (PDH) inhibition (Lam and Ko, 2012). It also displayed antiapoptotic effect on rat cortical neurons induced by Aβ in *in vitro* (Wang and Wang, 2009). In neuron-microglia cocultures, SchB exerted anti-neuroinflammatory activity by inhibiting MyD88/IKK/NF-κB signaling pathway, and the release of pro-inflammatory cytokines including NO, TNF-α, PGE2, IL-1β and IL-6 were all reduced. Moreover, SchB significantly inhibited ROS production and NADPH oxidase activity in microglia, thus playing a protective role in neurons (Zeng et al., 2012).

In the rat model, SchB might alleviate the damage of inflammatory response to nerve cells during cerebral ischemia-reperfusion via regulating the HSPA12B/PI3K/Akt signaling pathway (Jiang et al., 2016), indicating that SchB could inhibit the secondary inflammatory response after cerebral ischemia-reperfusion. This indicates that SchB might have a potential therapeutic effect on AD complicated with cerebral ischemia.

#### 3.1.5 Dibenzocyclooctene lignan-riched extract

Dibenzocyclooctene lignans are the characteristic compounds widely existed in *Schisandra.* It was shown that the extract from the fruits of *Schisandra chinensis*, which was enriched in dibenzocyclooctene lignans could alleviate the memory impairment in AD mice by inhibiting the activity of *β*-secretase in the cerebral cortex and hippocampus (Jeong et al., 2013). Wei et al. reported that the extract of *Schisandra chinensis* alleviated the inflammation caused by prostaglandins (PGs) metabolic disorders by regulating the metabolic disorders of polyunsaturated fatty acids in AD patients (Wei et al., 2019). These findings further suggest that dibenzocyclooctene lignans might be potential in preventing the occurrence or progression of AD.

### 3.2 Tetrahydrofuran lignans

Tetrahydrofuran lignans mainly exist in Camphoraceae, Magnoliaceae, Piperaceae, Cucurbitaceae, Nutmegaceae, Rehmanaceae, Compositae, Luteaceae, Lonicerae, Aristolaceae and other plants, which showed strong biological activities, including antitumor, antioxidant, anti-inflammatory, neuroprotective, insecticidal and estrogen-like effects as reported before (Briggs et al., 1997).

(−)-Talaumidin (**14**), isolated from the root of *Aristolochia arcuata*, has been shown to promote axon growth and displayed neuroprotection activity in primary rat cortical neurons and PC12 cells (Harada et al., 2015).

(−)-O-methylcubebin (**16**) and (−)-O-benzylcubebin (**17**), synthesized based on (−)-cubebin (**15**) from *Piper cubeba* L. f. in Piperaceae, showed the inhibitory effect on *P. gingivalis* (Rezende et al., 2016). Since the infection of bacteria would induce AD and the products of *P. gingivalis* could be detected in the brains, **15** might be useful in curing AD caused by infection.

### 3.3 Furofuranoid lignans

The structure of furofuranoid lignans was formed by condensation of hydroxyl groups on aliphatic hydrocarbon chains in tetrahydrofuran lignans. Some furofuranoid lignans including Syringaresinol (**18**), Pinoresinol (**19**), Sesamolin (**20**), Medioresinol (**21**) and so on were metabolized by intestinal flora to form enterolactone metabolites, which played a neuroprotective role after crossing the blood-brain barrier and reaching the brain. This provides evidence for lignans as potential modulators of the gut-brain axis (Senizza et al., 2020). (−)-7-epi-pinoresinol mr1 (**22**), (+)-medioresinol (**23**), and (+)-diapinoresinol (**24**) isolated from the leaves of *Eucommiae ulmoides* could protect the damage of PC-12 cells induced by H_2_O_2_ through PI3K/Akt/GSK-3β/NRF2 signaling pathway, which is one of the most important pathways in the regulation of Tau protein phosphorylation (Han et al., 2022).

Sesamin (SES) **(25)**, a major lignan that is mainly obtained from sesame seeds and oil (Andargie et al., 2021), which could cross the blood-brain barrier (BBB) and accumulate in the brain (Umeda-Sawada et al., 1999). Chondroitin sulfate proteoglycans (CSPGs) are an important component of glial scar, which usually formed after central nervous system (CNS) injury (Burda and Sofroniew, 2014). Increased CSPGs are mainly used as a chemical barrier to protects the CNS (Davies et al., 1997; Silver and Miller, 2004). SES was reported to increase the expression of CSPGs biosynthesis and decrease degradation-related genes in the hippocampus of LPS-treated mice (Yamada et al., 2022). The effects of SES on adult neurogenesis were more obvious in the dorsal hippocampus (cognitive center) than in the ventral hippocampus (emotional center) (Yamada et al., 2018). It was also found that SES could play a neuroprotective role in in streptozotocin (STZ)-induced diabetic rats. SES reduced anxiety/depression like behaviors, increased exercise/exploration activities, and improved passive avoidance learning and memory. It was suggested that SES could reduce blood glucose, inhibit neuroinflammation and enhance neurotrophic factors to against AD caused by diabetes (Ghaderi et al., 2021).

(−)-Sesamin **(26)** is a major lignan constituent in the roots of *Asiasarum sieboldi*, which ameliorated spatial and habit learning memory deficits by modulating both NMDA receptor and dopaminergic neuronl systems. After exposure to chronic electric footshock (EF)-induced stress, the levels of NMDA receptor phosphorylation were reduced by treatment with (−)-sesamin at both doses (25 and 50 mg/kg). The retention latency in passive avoidance test and dopamine levels in substantia nigra striatum were decreased by chronic EF stress, and increased after (−)-sesame treatment (Zhao et al., 2016).

### 3.4 Benzoxanthene lignans

Sauchinone **(27),** isolated from the roots of *Saururus chinensis*, supplied a useful adjunctive treatment for the treatmeng of AD cased by bacterial infection, since It enhanced phagocytosis of macrophages to *Escherichia coli* through p38/MAPK signaling pathway in rats macrophages in a concentration dependent manner (Jeong et al., 2014).

### 3.5 Norlignans

(*R*)-1-(3-methoxy-4-hydroxyphenyl)-2-(3-methoxy-1-hydroxypropylphenoxy)-3-hydroxypropan (**28**) and (*S*)-1-(3-methoxy-4-hydroxyphenyl)-2-(3-methoxy-1 - hydroxypropylphenoxy)-3-hydroxypropan (**29**) are a pair of enantiomers isolated from the seeds of *Prunus tomentosa* by Liu et al. (Liu et al., 2019)*.* They were reported to exhibit hydrogen bonding interactions with Aβ in molecular docking studies, sharing the similar binding site at residue Gln15 with the positive compound curcumin, and their activities on anti-Aβ aggregation were further verified by thioflavin T (ThT) method with the inhibitory rate 63.25 ± 2.68% (**28**) and 67.13 ± 0.90% (**29**), higher than positive drug.

### 3.6 Neolignans

Wang et al. isolated two rare 8′, 9′-dinor-3′, 7-epoxy-8, 4′-oxyneolignanes from the twigs and leaves of *Pithecellobium clypearia* named as (7*S*, 8*S*)- and (7*R*, 8*R*)-pithecellobiumin A (**30**, **31**) respectively, which are a pair of enantiomers of neolignans (Wang et al., 2018). Enantiomers compounds **30** (62.1%) and **31** (81.6%) showed different degrees of anti-Aβ aggregation activity by ThT method. However, they showed different interaction mode with Aβ according to molecular docking studies. Compound **30** interacted with Leu34 of Aβ while compound **31** interacted with Gly9 and Gln15. The results showed compounds **30** and **31** have potentials to treat AD by inhibiting the formation of Aβ aggregation.

## 4 Conclusion and Foresight

**FIGURE 3:**
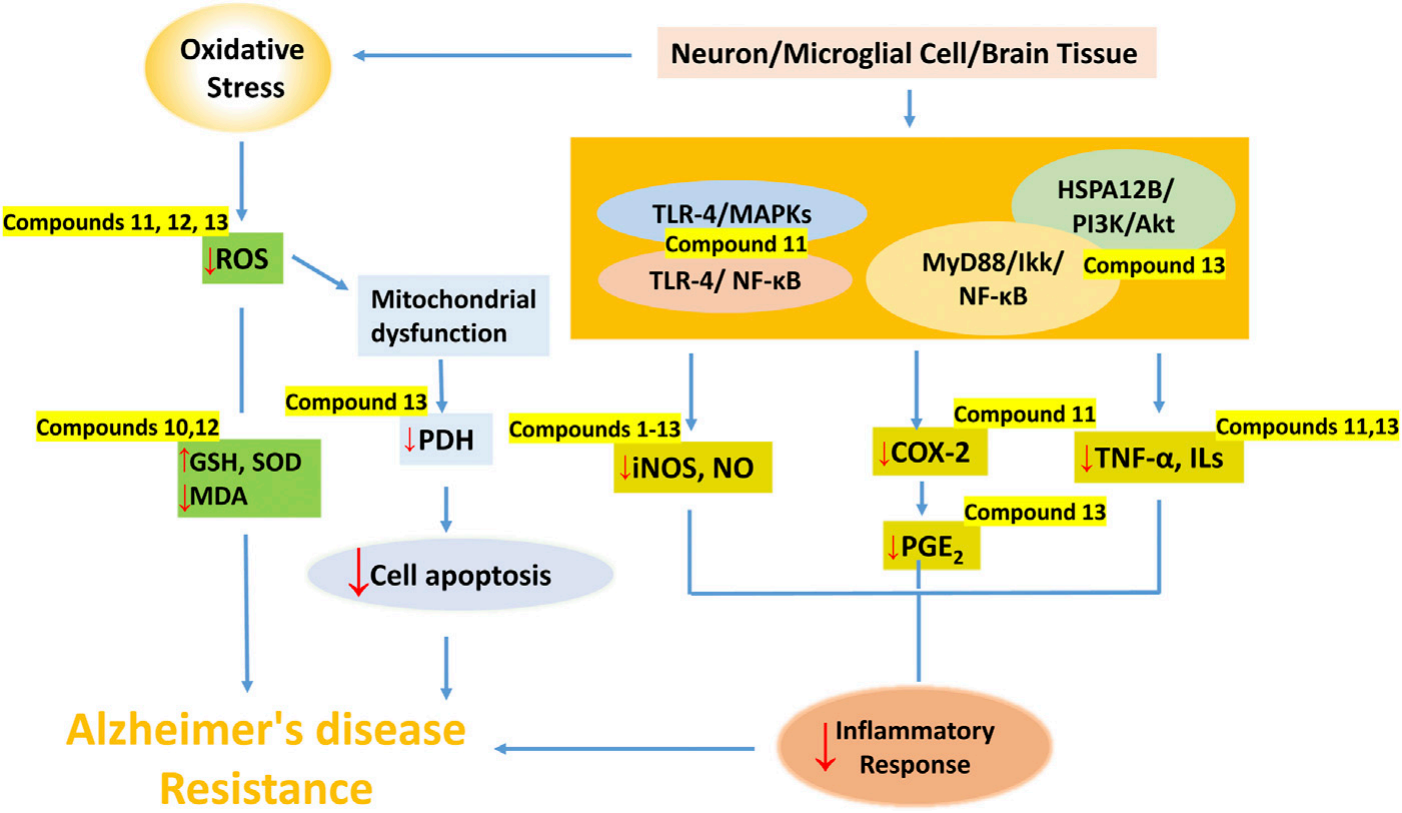
The main mechanisms of dibenzocyclooctene lignans against AD. (Dibenzocyclooctene lignans play a protective role in the nervous system through anti-inflammation, antioxidation and inhibition of neuronal apoptosis. Compounds **1**–**13** inhibited the activity of NO, compounds **11** and **13** inhibited the release of downstream inflammatory factors by regulating TLR-4-mediated NF-κB and MAPKs signaling pathways, MyD88/IKK/NF-κB and HSPA12B/PI3K/Akt signaling pathways. In addition, Compounds **10** and **12** up-regulated the expression of SOD and GSH, and inhibited MDA in brain tissue, while compounds **11** and **13** reduced the content of ROS to resist oxidative stress. Compound **13** also inhibited apoptosis by inhibiting JNK-mediated PDH inhibition.).

In this paper, we focus on the neuroprotective and cognitive enhancement effects of lignans and their mechanisms, providing a basis for the development of lignans as new anti-AD drugs. Lignans exerted neuroprotective and cognitive enhancement effects through regulating vascular disorders, anti-infection, anti-inflammation, anti-oxidation, anti-apoptosis, antagonizing NMDA receptor, suppressing AChE activity, improving gut microbiota, and regulating different signaling pathways as shown in Table 1. Among them, biphenyl cyclooctene lignans are the most potential lignans, in which anti-inflamation, anti-oxidation and antipoptosis effects on neurons, microglial or brain tissue were widely reported (Figure 3).

**TABLE 1:** The action mechanisms of major lignans.

Category	Compound Name	Origin	*In vitro*/*In vivo* models	Mechanism	Ref
Dibenzocyclooctene lignans	Schisanchinin A (**1**)	The fruits of *Schisandra chinensis*	LPS-induced BV2 microglia in mouse	Anti-inflammation	Hu et al. (2014)
	Schisanchinin B (**2**)				
	Deoxyschizandrin (**3**)				
	(±)-*γ*-schizandrin (**4**)				
	Gomisin G (**5**)				
	(-)-gomisin M_1_ (**6**)				
	(-)-gomisin L_1_ (**7**)				
	(+)-gomisin M_2_ (**8**)				
	(+)-gomisin K_3_ (**9**)				
	Schisandrin (**10**)				
	Gomisin A (**11**)				
	Schisandrin (**10**)	The fruits of *Schisandra chinensis*	Mice	Anti-oxidation	Cao et al. (2015)
	Gomisin A (**11**)	*The* fruits of *Schisandra chinensis*	LPS-stimulated N9 microglia	Anti-inflammation	Dong, Hung et al. (2006)
			LPS-stimulated N9 microglia	Anti-oxidation	Wang, Hu et al. (2014)
	Schisantherin A (**12**)	The fruits of *Schisandra chinensis*	Aβ induced AD rat	Anti-oxidation	Li, Zhao et al. (2014)
			SH-SY5Y cells induced by 6—OHDA	Anti-inflammation	Zhang, Lun et al. (2015)
	Schisandrin B (**13**)	The fruits of *Schisandra chinensis*	Scopolamine-induced dementia mice	Suppressing AChE activity	Giridharan, Thandavarayan et al. (2011)
			PC12 cells exposed to 3-NP	Anti-apoptotic	Lam and Ko (2012)
			Rat cortical neurons induced by Aβ *in vitro*	Anti-apoptotic	Wang and Wang (2009)
			Neuron–microglia co-cultures	Anti-oxidation	Zeng, Zhang et al. (2012)
				Anti-inflammation	
			Rat model of cerebral ischemia-reperfusion injury	Regulation of vascular disorders	Liu et al. (2019)
Tetrahydrofuran lignans	(−)-Talaumidin (**14**)	The roots of *Aristolochia contorta*	PC12 cells	Promoting neuronal growth	Harada, Kenichi et al. (2015)
	(−)-cubebin (**15**)	The synthesized compounds	*Porphyromonas gingivalis* suspension	Anti-infection	(Karen, C. et al., 2016)
	(−)-O-methylcubebin (**16**)				
	(−) -O-benzylcubebin (**17**)				
Furofuranoid lignans	Medioresinol (**18**)	Sesame seeds, Cloudberry	Intestinal flora	Modulation of gut microbiota	Senizza, Rocchetti et al. (2020)
	Syringaresinol (**19**)	Rye, whole Grain flour			
	Pinoresinol (**20**)	Olive oil			
	Sesamolin (**21**)	Sesame seeds			
	(–)-7-epi-Pinoresinol mr1 (**22**)	The leaves of *Eucommiae ulmoides*	H_2_O_2_-treated PC12 cells	Anti-Tau protein phosphorylation	Han, Yu et al. (2022)
	(+)-Medioresinol (**23**)			Antioxidant	
	(+)-Diapinoresinol (**24**)				
	Sesamin (**25**)	Sesame seeds and oil	LPS-treated mice	Promoting neuronal growth	Yamada, Maeda et al. (2022)
			Rat model of diabetes	Anti-diabetics	Ghaderi, Rashno et al. (2021)
				Anti-inflammation	
	(−)-Sesamin (**26**)	The roots of *Asiasarum sieboldi*	Mice induced by chronic electric footshock	Antagonizing NMDA receptor	Zhao, Shin et al. (2016)
Benzoxanthene lignans	Sauchinone (**27**)	The roots of *Saururus chinensis*	Mice injected with 4% Brewer thioglycollate	Anti- inflammation	Jeong, Choi et al. (2014)
Norlignans	(*R*)-1-(3-methoxy-4-hydroxyphenyl)-2-(3-methoxy-1-hydroxypropylphenoxy)-3-hydroxypropan (**28**)	The seeds of *Prunus tomentosa*	ThT method	Inhibition of Aβ aggregation	Liu et al. (2019)
	(*S*)-1-(3-methoxy-4-hydroxyphenyl)-2-(3-methoxy-1-hydroxypropylphenoxy)-3-hydroxypropan (**29**)				
Benzofuran lignans	(7*S*, 8*S*)-pithecellobiumin A (**30)**	The twigs and leaves of *Pithecellobium clypearia*	ThT method	Inhibition of Aβ aggregation	Wang, Zhou et al. (2018)
	(7*R*, 8*R*)-pithecellobiumin A (**31**)				

In addition, since long-term use of existing commercially available drugs will bring serious side effects, drugs with less side effects would be more popular in the future. As natural compounds, lignans are abundant in fruits, vegetables and grains with low toxicity and good bioavailability (Jin et al., 2013; Kumar et al., 2021)

One of the most challenging problems in the development of therapeutic drugs for neurodegenerative diseases is that drugs cannot cross the blood-brain barrier. However, the lignans such as schisantherin A and schisandrin B were reported to be capable to cross the BBB due to their lipid-soluble properties and small-molecular mass (Hu et al., 2012; Wang et al., 2018), which further improve the possibilities of lignans to be developed as anti-AD drugs.
